# The *DsbA-L* gene is associated with respiratory function of the elderly via its adiponectin multimeric or antioxidant properties

**DOI:** 10.1038/s41598-020-62872-5

**Published:** 2020-04-06

**Authors:** Kentaro Oniki, Hirofumi Nohara, Ryunosuke Nakashima, Yui Obata, Narumi Muto, Yuki Sakamoto, Keiko Ueno-Shuto, Tadashi Imafuku, Yu Ishima, Hiroshi Watanabe, Toru Maruyama, Koji Otake, Yasuhiro Ogata, Mary Ann Suico, Hirofumi Kai, Tsuyoshi Shuto, Junji Saruwatari

**Affiliations:** 1 Division of Pharmacology and Therapeutics, Graduate School of Pharmaceutical Sciences, Kumamoto University, Kumamoto, Japan; 2 Department of Molecular Medicine, Graduate School of Pharmaceutical Sciences, Kumamoto University, Kumamoto, Japan; 3 Laboratory of Pharmacology, Division of Life Science, Faculty of Pharmaceutical Sciences, Sojo University, Kumamoto, Japan; 4 Department of Biopharmaceutics, Graduate School of Pharmaceutical Sciences, Kumamoto University, Kumamoto, Japan; 5 Department of Pharmacokinetics and Biopharmaceutics, Institute of Biomedical Sciences, Tokushima University, Tokushima, Japan; 6 Japanese Red Cross Kumamoto Health Care Center, Kumamoto, Japan

**Keywords:** Chronic obstructive pulmonary disease, Translational research

## Abstract

Oxidative stress and inflammation play a key role in the age-related decline in the respiratory function. Adipokine in relation to the metabolic and inflammatory systems is attracting growing interest in the field of respiratory dysfunction. The present clinical and experimental studies investigated the role of the *disulfide bond-forming oxidoreductase A-like protein* (*DsbA-L*) gene, which has antioxidant and adiponectin multimeric (i.e. activation) properties, on the respiratory function of the elderly. We performed a retrospective longitudinal genotype-phenotype relationship analysis of 318 Japanese relatively elderly participants (mean age ± standard deviation: 67.0 ± 5.8 years) during a health screening program and an *in vitro DsbA-L* knock-down evaluation using 16HBE14o-cells, a commonly evaluated human airway epithelial cell line. The *DsbA-L* rs1917760 polymorphism was associated with a reduction in the ratio of forced expiratory volume in 1 second (FEV1)/forced vital capacity (FVC) and %FEV1 and with the elevation of the prevalence of FEV1/FVC < 70%. We also confirmed that the polymorphism was associated with a decreased respiratory function in relation to a decrease in the ratio of high-molecular-weight adiponectin/total adiponectin (as a marker of adiponectin multimerization) and an increase in the oxidized human serum albumin (as an oxidative stress marker). Furthermore, we clarified that *DsbA-L* knock-down induced oxidative stress and up-regulated the mucus production in human airway epithelial cells. These findings suggest that the *DsbA-L* gene may play a role in protecting the respiratory function of the elderly, possibly *via* increased systemic adiponectin functions secreted from adipocytes or through systemic and/or local pulmonary antioxidant properties.

## Introduction

Airway inflammation and an impaired lung function are common in elderly populations, leading to decreased quality of life and increase in morbidity and mortality^1–3^. Although exposures to cigarette smoke and other environmental pollutants (*e.g.* biomass fuel exposure and air pollution) are major risk factors for the development and progression of respiratory dysfunction^4,5^, previous evidence has shown that age-related changes in the respiratory functions are also key factors for respiratory dysfunction^1–3^. Aging causes the loss of lung elasticity, decreased respiratory muscles, and a decreased surface area for alveolar gas exchange, leading to an impaired respiratory function^1–3^. Furthermore, local and/or systemic oxidative stress as well as inflammation play substantial roles in the age-related decline in the respiratory function^1,2^. However, these changes in respiratory pathological features with aging are affected by many other factors, including not only environmental toxins but also genetic predisposition, metabolic status and oxidative stress, and are thus considered to be complex and interactive^1–3^.

Adiponectin (APN) is an adipose tissue-derived adipocytokine that is abundant in plasma and has anti-inflammatory, anti-diabetic and anti-atherogenic properties^6–8^. Previous clinical trials have indicated that the APN level is closely related to the respiratory function parameters^9^. APN knock-out mice were reported to show abnormal alveolarization and increases in both proteolytic and tumor necrosis factor-alpha (TNF-α) activity, which resulted in an emphysematous phenotype with an inflammatory condition^10^. APN decreases the production and activity of inflammatory cytokines, including TNF-α and interleukin-6, and increases the production of anti-inflammatory cytokines in epithelial cells, monocytes and macrophages^9^.

Plasma APN exists in three major oligomeric complexes: trimer and hexamer forms and a high-molecular-weight (HMW) oligomer that is the major relevant form for improving insulin sensitivity, anti-inflammatory and antidiabetic activities. Notably, low levels of the HMW oligomer are an independent risk factor for several metabolic diseases^11,12^. Disulfide bond-forming oxidoreductase A-Like protein (DsbA-L) is highly expressed in the endoplasmic reticulum (ER) and mitochondria and was renamed from glutathione *S*-transferase (GST) kappa 1^13,14^. DsbA-L is involved in cell detoxification of xenobiotics, endogenous toxic metabolites and free radicals, including toxic metabolites contained in cigarette smoke^13^. DsbA-L is also reported to be an important regulator of APN multimerization in 3T3-L1 cells^14^ and humans *in vivo*^15^. Therefore, the *DsbA-L* gene may affect the decline in the respiratory function, but the details remain unclear.

A common polymorphism in the human *DsbA-L* gene at -1308 bp (rs1917760) has been identified and can influence the DsbA-L expression and activity^16^. The allele frequency of the rs1917760 polymorphism is reported to be approximately 20% in Asians^16^. Gao *et al*. previously showed that the *DsbA-L* rs1917760 polymorphism was associated with increased insulin secretion and fat deposition in a cross-sectional study^17^. Our recent clinical investigation revealed that the *DsbA-L* rs1917760 polymorphism is associated with an increased body mass index (BMI) in relation to decreasing the ratio of HMW/total APN^15^.

In the present study, we performed a clinical pharmacogenomics analysis and an *in vitro* evaluation to investigate the role of the *DsbA-L* gene in the respiratory dysfunction of the elderly. The present findings of this study provide insight into potentially novel preventive or therapeutic targets for the age-related decline in the respiratory function, which leads to a decreased quality of life and increase in morbidity and mortality in elderly individuals^1–3^.

## Results

### Association between the *DsbA-L* genotype and the respiratory function

**Figure 1:**
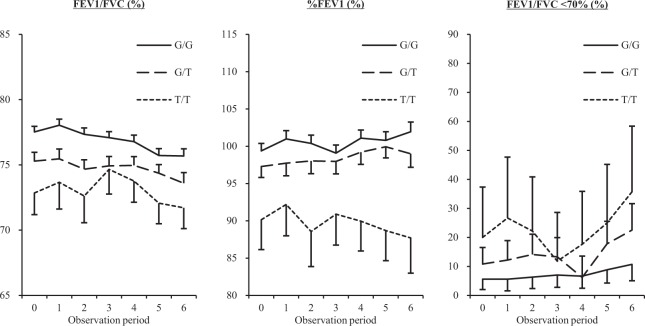
Longitudinal differences of the mean values of FEV1/FVC and %FEV1 and prevalence of FEV1/FVC < 70% between the *DsbA-L* genotypes. The mean values of FEV1/FVC and %FEV1 and the prevalence of FEV1/FVC < 70% are shown as dashed-dotted, dotted, and solid lines for the subjects with the *DsbA-L* G/G, G/T and T/T genotypes, respectively. The bars represent the standard errors or 95% confidence interval. FEV1, forced expiratory volume in 1 second; FVC, forced vital capacity ratio; DsbA-L, disulfide bond-forming oxidoreductase A-Like protein.

The study subjects included in this study were relatively elderly individuals (mean age ± standard deviation: 67.0 ± 5.8). The demographic characteristics at baseline of the subjects are shown in Table 1. The genotype frequencies of the *DsbA-L* rs1917760 G/G, G/T and T/T were 56.1%, 37.8% and 6.1%, respectively. The carriers of the *DsbA-L* T/T genotype had lower values of forced expiratory volume in 1 second (FEV1)/forced vital capacity (FVC) and %FEV1, a higher prevalence of FEV1/FVC < 70% and a greater proportion of overweight than those with the G/G or G/T genotype at baseline (Table 1). The longitudinal differences in the values of FEV1/FVC and %FEV1 and the prevalence of FEV1/FVC < 70% among the *DsbA-L* genotypes are shown in Fig. 1. FEV1/FVC and %FEV1 values differed among the *DsbA-L* genotypes through the observation period (Fig. 1). The influence of the *DsbA-L* genotype on the respiratory function was also analyzed using multiple regression models adjusted by age, gender, BMI and smoking status based on the generalized estimating equations approach (Table 2). The longitudinal values of FEV1/FVC and %FEV1 and the prevalence of FEV1/FVC < 70% were found to be significantly higher in the carriers of the *DsbA-L* T/T and/or G/T genotypes than in those with the G/G genotype independently of age, gender, BMI and smoking status (Table 2). Since the *DsbA-L* rs1917760 polymorphism was significantly associated with an overweight status^15^, we performed stratified multivariable analyses by the presence of an overweight status (Supplementary Table 2). As a result, the effects of *DsbA-L* rs1917760 polymorphism on the reduction in the respiratory function were found to be more pronounced in overweight subjects than in normal-weight subjects (Supplementary Table 2).

**Table 1:** Clinical characteristics stratified by the *DsbA-L* genotype at baseline.

	*DsbA-L* genotype			
	G/G (n = 178)	G/T (n = 120)	T/T (n = 20)	*P*
Female (%)	68 (38.2)	51 (42.5)	7 (35.0)	0.689
Age (years)	67.3 ± 5.9	66.4 ± 5.6	67.3 ± 6.0	0.473
BMI (kg/m^2^)	22.7 ± 2.9	22.6 ± 2.5	23.9 ± 2.2	0.143
FEV1 / FVC (%)	77.5 ± 5.7	75.3 ± 7.3	72.9 ± 7.5	0.001
%FEV1 (%)	99.4 ± 13.4	97.3 ± 16.1	90.2 ± 17.9	0.025
%FVC (%)	108.1 ± 13.6	109.6 ± 14.7	104.5 ± 14.7	0.286
Fasting blood glucose (mg/dL)	100.3 ± 19.7	102.4 ± 19.6	103.8 ± 7.9	0.570
Systolic BP (mmHg)	124.1 ± 18.1	122.7 ± 17.8	121.1 ± 12.9	0.580
Diastolic BP (mmHg)	72.6 ± 10.5	71.4 ± 11.8	73.0 ± 11.4	0.623
LDL-C (mg/dL)	124.8 ± 27.8	123.5 ± 28.0	115.9 ± 25.3	0.390
HDL-C (mg/dL)	69.8 ± 16.6	69.6 ± 16.0	70.9 ± 23.9	0.956
TG (mg/dL)	101.0 ± 53.2	102.9 ± 46.4	98.1 ± 45.2	0.907
FEV1/FVC < 70% (%)	10 (5.6)	13 (10.8)	4 (20.0)	0.046
Overweight (%)	87 (48.9)	54 (45.0)	15 (75.0)	0.046
Diabetes (%)	20 (11.2)	13 (10.8)	2 (10.0)	0.983
Hypertension (%)	75 (42.1)	47 (39.2)	9 (45.0)	0.824
Dyslipidemia (%)	80 (44.9)	55 (45.8)	7 (35.0)	0.661
Ever-smoking status (%)	69 (38.8)	63 (52.5)	10 (50.0)	0.135
Alcohol intake (g/day)	7.7 ± 11.7	7.9 ± 12.8	5.1 ± 8.0	0.619

The data are the means ± standard deviation, medians (range) or proportions for categorical variables. DsbA-L, disulfide bond-forming oxidoreductase A-like protein; BMI, body mass index; FEV1: percent predicted forced expiratory volume in 1 second; FVC, percent predicted forced vital capacity; BP, blood pressure; LDL-C, low-density lipoprotein cholesterol; HDL, high-density lipoprotein cholesterol; TG, triglyceride.

**Table 2:** Longitudinal associations of the *DsbA-L* genotype with the values of FEV1/FVC and %FEV1 and the prevalence of FEV1/FVC < 70% in the multivariable regression models based on the generalized estimating equations approach.

*DsbA-L* genotype	FEV1/FVC (%)			%FEV1 (%)			FEV1/FVC < 70%		
	B*	SE	*P*	B*	SE	*P*	OR*	95% CI	*P*
G/G	0			0			1		
G/T	−2.393	0.696	<0.001^†^	−2.073	1.724	0.229	2.608	1.240–5.484	0.011^†^
T/T	−3.492	1.554	0.025	−9.223	3.697	0.013^†^	3.715	1.070–12.896	0.039
G/G	0			0			1		
G/T or T/T	−2.552	0.661	<0.001^†^	−3.105	1.645	0.059	2.767	1.355–5.650	0.005^†^

*Adjusted by age, gender, BMI, smoking status. ^†^Statistical significance remained following the use of Bonferroni’s correction. DsbA-L, disulfide bond-forming oxidoreductase A-like protein; BMI, body mass index; %FEV1, percent predicted forced expiratory volume in 1 second; OR, odds ratio; CI, confidence interval; SE, standard error.

### Association between the *DsbA-L* genotype, APN levels and the respiratory function

**Figure 2:**
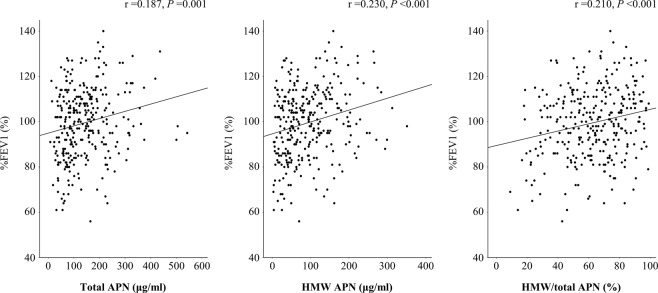
The correlations of %FEV1 with the total, HMW APN and the ratio of HMW/total APN. Correlation coefficients (r) and P values were calculated by Pearson’s correlation test. FEV1, forced expiratory volume in 1 second; HMW, high-molecular-weight; APN, adiponectin.

The mean values ± standard deviation of total and HMW APN and the ratio of HMW/total APN (*i.e.* index of APN multimerization) were 141.0 ± 89.9 ng/mL, 92.4 ± 67.3 ng/mL and 62.6% ± 20.3%, respectively. The value of %FEV1 was positively correlated with the values of total and HMW APN and the ratio of HMW/total APN (r = 0.187, *P* = 0.001; r = 0.230, *P* < 0.001; r = 0.210, *P* = 0.002, respectively) (Fig. 2). In contrast, the value of FEV1/FVC was not correlated with the values of total or HMW APN or the ratio of HMW/total APN (r = 0.081, *P* = 0.156; r = 0.073, *P* = 0.202; r = −0.032, *P* = 0.577, respectively). Our recent study showed that the ratio of HMW/total APN in carriers of the *DsbA-L* T/T genotype was lower than in those with the G/G or G/T genotype among almost similar subjects^15^. Furthermore, the ratio of HMW/total APN was significantly lower in overweight subjects with the T/T genotype than in normal-weight subjects with the G/G genotype^15^. We assessed the effect of the *DsbA-L* T allele on APN multimerization in the present study but failed to detect any significant association between the *DsbA-L* T allele and the ratio of HMW/total APN (Table 3).

**Table 3:** Associations of the *DsbA-L* T allele with the HMW/total APN (%) and the oxidized HSA (%) in the multiple regression models.

*DsbA-L* genotype	HMW/total APN (%)			Oxidized HSA (%)		
	B*	SE	*P*	B*	SE	*P*
G/G	0			0		
G/T or T/T	−0.360	2.276	0.874	1.106	0.395	0.005

*Adjusted by age, gender, BMI and smoking status. DsbA-L, disulfide bond-forming oxidoreductase A-like protein; HMW, high molecular weight; APN, adiponectin; HSA, human serum albumin; BMI, body mass index; B, partial regression coefficient; SE, standard error.

### Association between the *DsbA-L* genotype, oxidative stress and the respiratory function

The mean values ± standard deviation of oxidized human serum albumin (HSA) (as a systemic oxidative stress marker) was 44.9% ± 3.6%. The oxidized HSA was not correlated with the value of %FEV1 (r = −0.013, *P* = 0.817) or FEV1/FVC (r = −0.013, *P* = 0.821). We previously reported that the oxidized HSA tended to be higher in carriers of the *DsbA-L* G/T or T/T genotypes than in those with the G/G genotype among similar subject^15^. In the present study, a multivariable analysis showed that oxidized HSA levels were significantly higher in the carriers of the *DsbA-L* T allele than in those with the G/G genotype, independent of the BMI (Table 3).

### Possible influence of the *DsbA-L* genotype on the respiratory function, APN multimerization and oxidative stress: a pathway analysis

**Figure 3:**
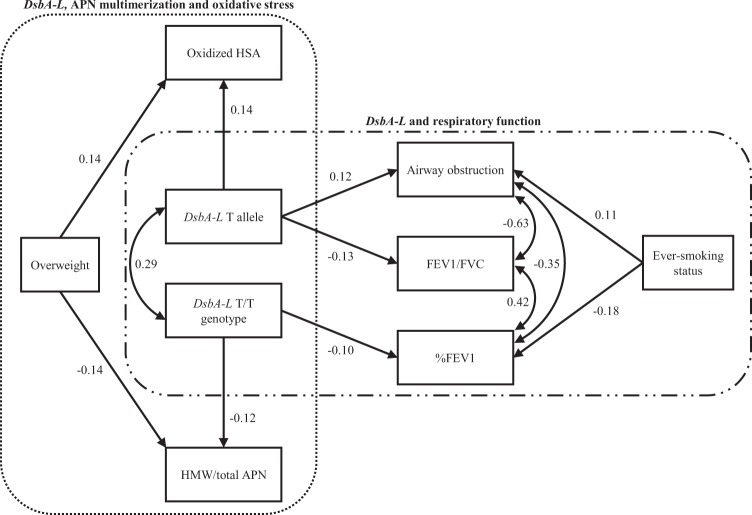
The structural equation modeling diagram of the *DsbA-L* genotype and respiratory function. Lines with numbers indicate significant paths with standardized β coefficients (*P* < 0.05). FEV1, forced expiratory volume in 1 second; DsbA-L, disulfide bond-forming oxidoreductase A-Like protein; HMW, high-molecular-weight; BMI, body mass index.

To verify the observed associations of the *DsbA-L* T/T genotype or T allele with the respiratory function, APN multimerization and oxidative stress, we performed a pathway analysis using structural equation modeling incorporating other potential risk factors for respiratory dysfunction (*e.g.* ever-smoking status) (Fig. 3). The fitness statistics goodness of fit index (GFI), adjusted GFI (AGFI), and root mean square error of approximation (RMSEA) were 0.987, 0.968 and 0.006, respectively, indicating a good fit for the structural equation model. Moreover, all 1000 bootstrap runs exhibited successful minimization and were included in the bootstrap analysis. The result of the bootstrap evaluation for this structural equation model showed that the mean values, SEs and 95% CIs for all covariates obtained using the bootstrap analysis were generally comparable to the estimates obtained using the structural equation modeling (Supplementary Table S1). The *DsbA-L* T/T genotype or T allele appears to influence the decreased respiratory function (*i.e.* reduction in FEV1/FVC and %FEV1 and increase in the risk of FEV1/FVC < 70%) (Fig. 3). Furthermore, the *DsbA-L* T/T genotype or T allele was also associated with a reduction in the ratio of the HMW/total APN and elevation in the oxidized HSA level (Fig. 3). In contrast, an overweight status was independently associated with a reduction in the ratio of the HMW/total APN and elevation in the oxidized HSA level (Fig. 3). Moreover, an ever-smoking status was also independently associated with a decreased respiratory function (Fig. 3).

### Induction of oxidative stress due to *DsbA-L* knock-down in human airway epithelial cells

**Figure 4:**
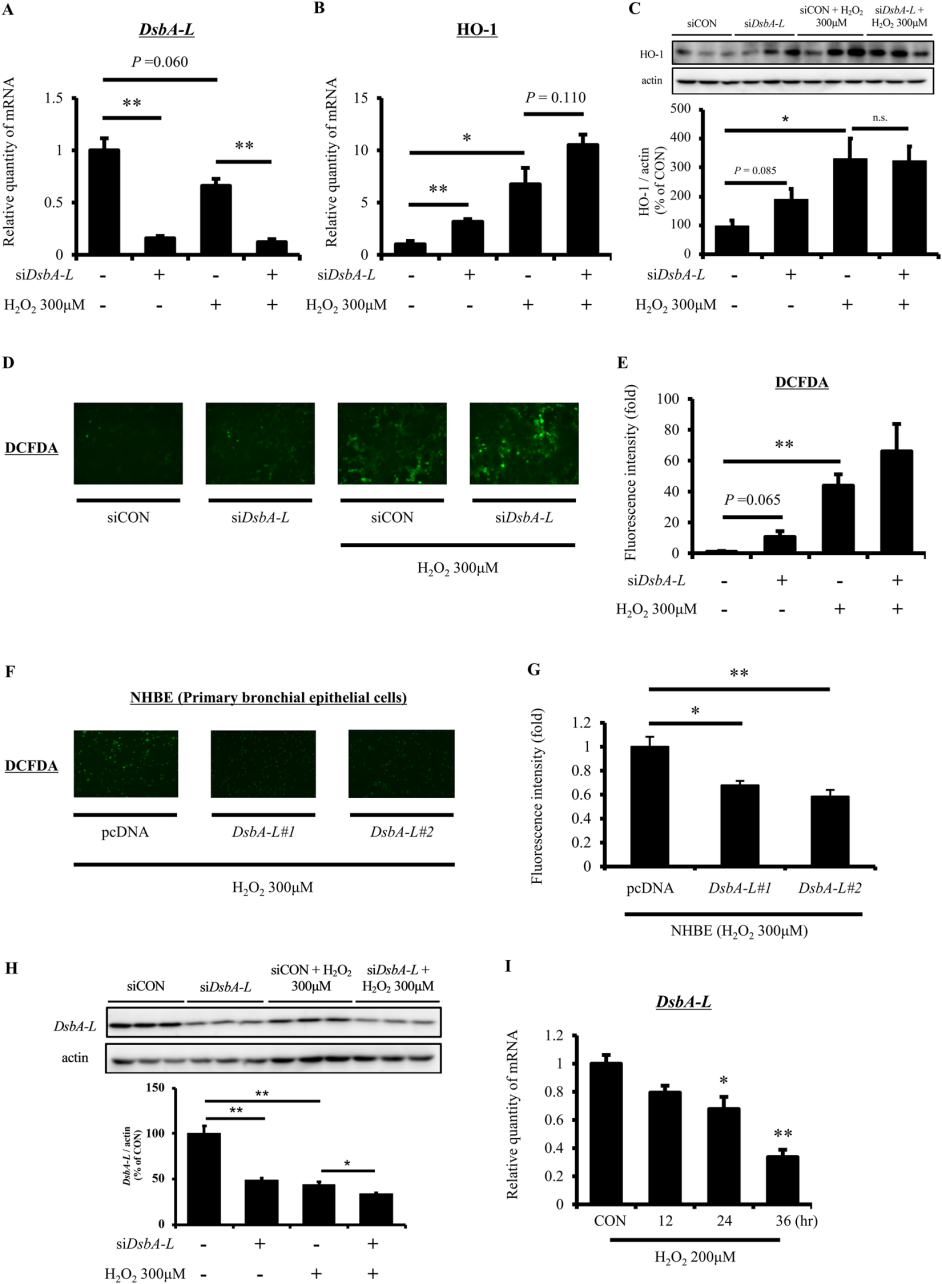
*DsbA-L* knock-down induces oxidative stress in human airway epithelial cells. (**A,B**) 16HBE14o- cells were transfected with control or *DsbA-L*-specific siRNA (50 nM) for 48 hr. After preincubation, cells were incubated with H_2_O_2_ (300 μM) for 6 hr and then quantitative real-time RT-PCR was performed using isolated RNA to determine the level of indicated genes. 18srRNA was used as internal control. (**C**) Immunoblotting with HO-1 antibody was performed with the lysates of siRNA-treated (50 nM) and/or H_2_O_2_-incubated (300 μM, 6 hr) 16HBE14o- cells. The band intensity was quantified by Multi Gauge software. (**D,E**) DCFDA staining was performed and analyzed with fluorescence microscopy in siRNA-treated (50 nM) and/or H_2_O_2_-incubated (300 μM, 1 hr) 16HBE14o- cells. (**F,G**) DCFDA staining was performed and analyzed with fluorescence microscopy in two lots of *DsbA-L*-transfected under H_2_O_2_-incubated (300 μM, 1 hr) primary NHBE cells. (**H**) Immunoblotting with *DsbA-L* antibody was performed with the lysates of siRNA-treated (50 nM) and/or H_2_O_2_-incubated (300 μM, 6 hr) 16HBE14o- cells. The band intensity was quantified by Multi Gauge software. (**I**) 16HBE14o- cells were treated with H_2_O_2_ (200 μM) for 12, 24 and 36 hr and then quantitative real-time RT-PCR was performed using isolated RNA to determine the level of *DsbA-L* gene. 18srRNA was used as internal control. Full unedited gel was shown in Supplementary Fig. S1. DsbA-L, disulfide bond-forming oxidoreductase A-Like protein; DCFDA, 2′,7′-dichlorodihydro-fluorescein diacetate acetyl ester. Data are means ± standard error; n = 3 / group. *P* values were assessed by Student’s t test (*P < 0.05, **P < 0.01, ***P < 0.001).

DsbA-L is expressed in mitochondria of systemic tissues, including lung tissue^18^. Given that *DsbA-L* polymorphism affects the decline in the respiratory function according to the results of our human *in vivo* studies (Figs. 1 and 3), there was a possibility that the *DsbA-L* might also exert a protective role in lung tissue. To explore the potential protective mechanisms of *DsbA-L* against respiratory dysfunction, we first analyzed the oxidative stress under the conditions of *DsbA-L* knock-down and/or treatment with H_2_O_2_ (300 μM) in human airway epithelial cells (Fig. 4). *DsbA-L* knock-down significantly decreased the *DsbA-L* expression (Fig. 4A). *Heme oxygenase* (*HO*)*-1*, an enzyme that catalyzes the degradation of heme, was increased under both *DsbA-L* knock-down and H_2_O_2_ treatment conditions at both mRNA and protein levels (Fig. 4B,C). In addition, the mRNA level of *HO-1* increased additively under conditions of both *DsbA-L* knock-down and H_2_O_2_ treatment (Fig. 4B). Furthermore, the elevation of ROS due to *DsbA-L* knock-down and H_2_O_2_ treatment was also detected with DCFDA (Fig. 4D,E). Consistently, the reduction of H_2_O_2_-induced ROS due to *DsbA-L* overexpression was detected with DCFDA (Fig. 4F,G). However, treatment with H_2_O_2_ decreased the *DsbA-L* mRNA and protein expression, and the *DsbA-L* mRNA-lowering effect by H_2_O_2_ became more pronounced with time (Fig. 4H,I).

### Up-regulation of mucus production due to *DsbA-L* knock-down in human airway epithelial cells

**Figure 5:**
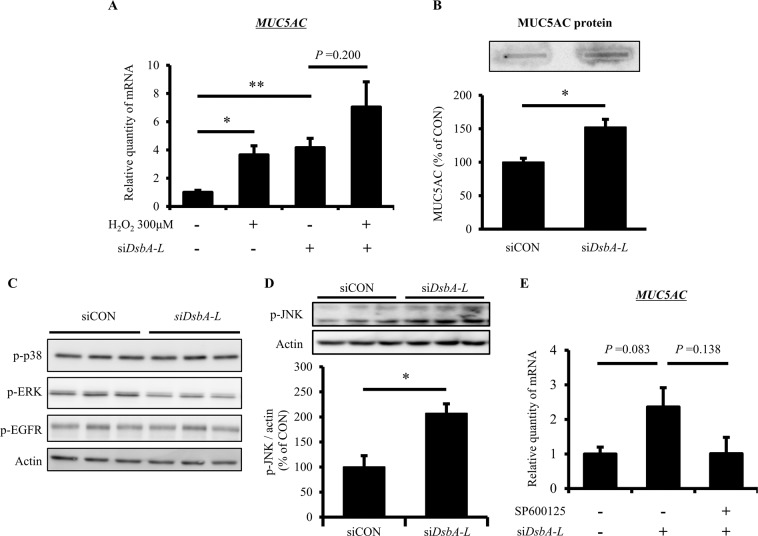
*DsbA-L* knock-down up-regulates mucus production through JNK signaling activation in human airway epithelial cells. (**A**) 16HBE14o- cells were transfected with control or *DsbA-L-*specific siRNA (50 nM) for 48 hr. After preincubation, cells were incubated with H_2_O_2_ (300 μM) for 6 hr and then quantitative real-time RT-PCR was performed using isolated RNA to determine the level of *MUC5AC* gene. (**B**) Condition medium from 16HBE14o- cells transfected with or without *DsbA-L-*specific siRNA (50 nM) for 48 hr were subjected to Slot-blot analysis to detected MUC5AC protein. The band intensity was quantified by Multi Gauge software. (**C,D**) Immunoblotting with indicated antibodies were performed with the lysates of siRNA-treated (50 nM, 48 hr) 16HBE14o- cells. The band intensity was quantified by Multi Gauge software. (**E**) *MUC5AC* gene expression of SP600125 (20 μM, 6 hr)-treated 16HBE14o- cells transfected with or without *DsbA-L*-specific siRNA (50 nM, 48 hr) were measured by quantitative real-time RT-PCR. 18srRNA was used as internal control. Full unedited gels were shown in Supplementary Figs. S2–S5. DsbA-L, disulfide bond-forming oxidoreductase A-Like protein; JNK, c-jun N-terminal kinase. Data are means ± standard error; n = 3 / group. *P* values were assessed by Student’s t test (*P < 0.05, **P < 0.01).

In order to assess whether or not the *DsbA-L* gene has a protective role in the accumulation of mucus in airway epithelial cells, we analyzed the effect of *DsbA-L* knock-down on the expression of *MUC5AC* as a mucus gene marker (Fig. 5). In addition, we investigated whether or not c-jun N-terminal kinase (JNK) acts as a regulator for *DsbA-L* knock-down-induced *MUC5AC* up-regulation. Both *DsbA-L* knock-down and treatment with H_2_O_2_ (300 μM) increased the level of *MUC5AC* mRNA, and the combination of *DsbA-L* knock down and H_2_O_2_ treatment additively increased the level of *MUC5AC* mRNA (Fig. 5A). Furthermore, *DsbA-L* knock-down increased the level of MUC5AC protein and p-JNK/Actin (Fig. 5B–D), and elevation of the level of *MUC5AC* mRNA due to *DsbA-L* knock-down was attenuated by treatment with SP600125, which is a potent, selective and reversible inhibitor of JNK (Fig. 5E).

## Discussion

**Figure 6:**
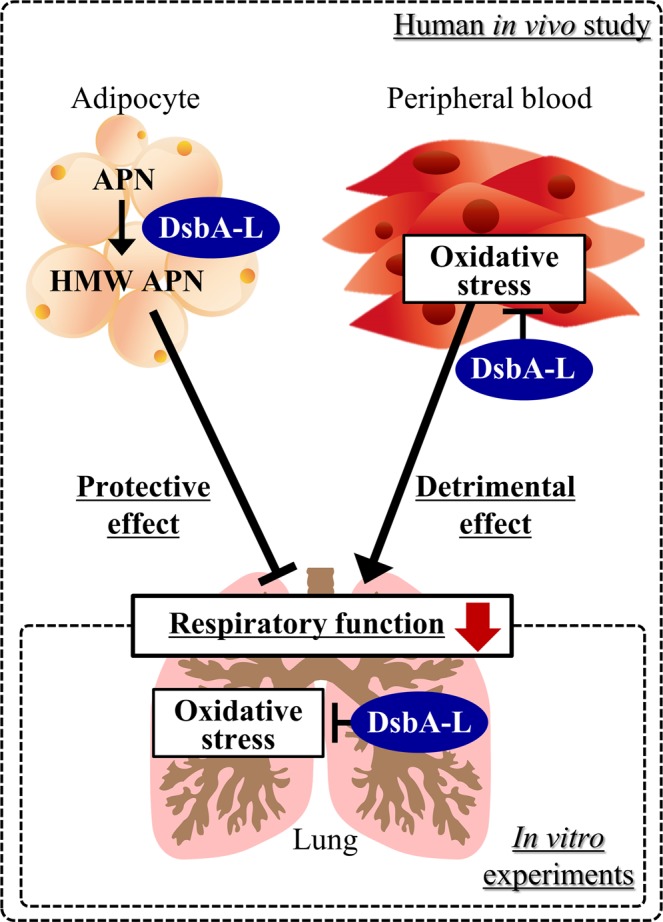
Proposed mechanisms by which DsbA-L protects the age-related decline in the respiratory function. DsbA-L, disulfide bond-forming oxidoreductase A-Like protein; HMW, high-molecular-weight; APN, adiponectin.

This is the first study to show the influence of the *DsbA-L* gene on the respiratory function by both clinical and experimental procedures. In the present human *in vivo* study conducted in the Japanese elderly population, the *DsbA-L* rs1917760 polymorphism was involved in the reduction in the respiratory function in conjugation with a decrease in the ratio of HMW/total APN and an increase in the oxidized HSA level. The present experimental study determined that *DsbA-L* knock-down induced oxidative stress and up-regulated the mucus production in human airway epithelial cells. These findings suggest that the *DsbA-L* gene may have a role in protecting the respiratory function of the elderly, possibly through increased systemic functions of APN secreted from adipocytes or through systemic and/or pulmonary antioxidant properties (Fig. 6). The findings of this study implied the potential utility of upregulating *DsbA-L* (*e.g.* weight reduction, smoking cessation and administration of an inducer of DsbA-L^14^) as a potential preventive or therapeutic approach for combatting the age-related decline in the respiratory function.

Although the role of APN in lung tissues is not entirely clear, the association of APN levels with the decline in the respiratory function has been highlighted^9,19–21^. APN, especially HMW APN, has anti-inflammatory and protective properties for endothelial functions, which are mainly regulated by suppressing TNF-α production, acting on macrophages to promote the removal of early apoptotic cells and inducing the production of anti-inflammatory cytokines^9^. In humans, the mRNA level of *DsbA-L* in adipocytes was shown to be inversely correlated with the BMI^14^, and the *DsbA-L* rs1917760 polymorphism was associated with increased insulin secretion and fat deposition^17^. The findings of our previous study indicated that the influence of the *DsbA-L* rs1917760 polymorphism on APN multimerization was more pronounced in overweight subjects than in normal-weight subjects^15^. We therefore speculated that the effect of the *DsbA-L* T/T genotype on the respiratory function might be associated with differences in subjects’ weight status in relation to low APN multimerization.

The cGAS-cGAMP-STING pathway was recently found as a cytosolic DNA sensor of pathogen-derived DNA to mediate the innate immune response^22^. Obesity-induced mtDNA was reported to be associated with inflammatory responses due to the activation of the cGAS-cGAMP-STING pathway^23^. Furthermore, the *DsbA-L* gene was shown to suppress obesity-induced inflammation and insulin resistance by inactivating the cGAS-cGAMP-STING pathway^23^. In the present study, the multivariable analyses showed that the *DsbA-L* rs1917760 polymorphism was associated with a reduction in the respiratory function, even after adjusting for the BMI (Table 2), and this association was more pronounced in overweight subjects than in normal-weight subjects (Supplementary Table 2). These results suggest that the protective role of *DsbA-L* against the reduction in the respiratory function would be more significant in overweight subjects than in normal-weight subjects. Therefore, weight reduction in overweight patients with the *DsbA-L* T/T genotype carriers may be a simple and effective preventive strategy against the age-related reduction in the respiratory function, although further interventional studies will be needed.

Nakanishi *et al*. reported that APN knock-out mice showed an advanced respiratory dysfunction phenotype and extrapulmonary effects (*e.g.* fat atrophy, body weight loss, systemic inflammation and osteoporosis), and APN injection was able to suppress the development of respiratory dysfunction^10^. Another study of rats showed that the decline in the respiratory function was ameliorated by the administration of APN through the inhibition of ER stress-induced airway epithelial apoptosis^24^. In our present human study, the levels of total and HMW APN and the ratio of HMW/total APN correlated positively with the value of %FEV1 (Fig. 2), suggesting that APN might exert a protective role against the respiratory dysfunction. In contrast, plasma HMW APN may also reflect the inflammatory status^9^ or weight status^25^, rather than the respiratory function, in patients with advanced respiratory dysfunction. Therefore, further longitudinal clinical investigations evaluating the influence of APN multimerization on the development and progression of respiratory dysfunctions, while also paying careful attention to the weight status, in both healthy subjects and patients with various disease states of respiratory dysfunction are needed.

Substantial evidence suggests that increased oxidative stress is a potential pathogenetic mechanism underlying the age-related decline in the respiratory function, and elderly individuals with respiratory dysfunction are considered to be in a state of oxidative stress^1,2^. DsbA-L is also known as GSTK1^13,14^ and not only plays a role in APN multimerization but also has activities against a number of substrates associated with oxidative stress (*e.g.* l-chloro-2,4-dinitrobenzene, ethacrynic acid, cumene hydroperoxide and *t*-butyl hydroperoxide)^13^. A previous study showed that the knock-down of the *DsbA-L* gene in *Caenorhabditis elegans* resulted in a significant decrease in the respiration rate and a change in the fatty acid metabolism in mitochondria^26^. The present human *in vivo* study also showed that the *DsbA-L* T allele was associated with increased oxidized HSA levels (Table 3). In addition, we showed that the *DsbA-L* gene knock-down directly induced oxidative stress in human airway epithelial cells (Fig. 4). Therefore, the *DsbA-L* gene may play an important role in protection against oxidative stress and inflammation, possibly by affecting the cellular properties of detoxification of xenobiotics, endogenous toxic metabolites and/or free radicals in airway epithelial cells.

Airway mucus hypersecretion is a frequent symptom associated with decline in the respiratory function^27^. Inflammatory reactions, oxidative stress and viral or bacterial infection often cause mucus production, which is closely related to poor mucociliary clearance, airway occlusion, reduced peak expiratory flow and respiratory muscle weakness^28^. The ROS-activated JNK signaling pathway was reported to be a major mechanism against increased mucus production by cigarette smoking^29^. In this study, we showed that the knock-down of the *DsbA-L* gene up-regulated mucus production and activated the JNK signaling pathway in human airway epithelial cells (Fig. 5). Consistently, Chen *et al*. reported that the deficiency of DsbA-L is associated with impairment of the maximum respiratory capacity, elevated cellular oxidative stress and increased JNK activity^30^. Thus, the antioxidant effect of DsbA-L may act protectively against the decline in the respiratory function, partially through the deactivated JNK signaling pathway in lung tissue.

Several limitations associated with the present study should be noted. First, the human study is a retrospective design and was performed in a relatively small number of subjects. In particular, there were few subjects with the *DsbA-L* T/T genotype (n = 20), so further investigations with a larger number of subjects are needed. Second, bronchodilators were not used before lung function tests were conducted, so the respiratory function should be evaluated more accurately using bronchodilators. Third, the present human *in vivo* study showed the systemic role of *DsbA-L* in the respiratory function of the elderly, and the present *in vitro* study showed the local role of *DsbA-L* in lung tissue. However, we were unable to examine whether or not the *DsbA-L* rs1917760 polymorphism affects the lung expression of *DsbA-L* in this study. In addition, we were unable to assess whether or not individuals with the *DsbA-L* rs1917760 TT genotype also had an increased sputum production or increased frequency of bronchitis. Therefore, whether or not a relationship truly exists between the results of the human *in vivo* study and the *in vitro* study is unclear. Further investigations are needed to determine whether or not the results of the human *in vivo* study are linked to those in the *in vitro* study. Finally, although our *in vitro* study revealed the connection between loss of *DsbA-L* and up-regulation of *MUC5AC* in airway epithelial cell line 16HBE14o- cells both at mRNA and protein levels (Fig. 5A,B). However, to make the finding more confident, further analysis such as western blotting or ELISA rather than dot-blot-based method to detect MUC5AC protein may be required. We have extensively performed western blotting and ELISA, but have never been able to detect MUC5AC protein in 16HBE14o- cells unfortunately (data not shown). Future experiments using primary airway epithelial cells may help to show physiological relevance of MUC5AC up-regulation under the condition with DsbA-L down-regulation.

## Conclusion

The current human *in vivo* study revealed the potential association of the *DsbA-L* gene with the respiratory function from the perspective of systemic APN multimerization and the oxidative stress state in a relatively healthy elderly population (Fig. 6). In addition, our *in vitro* investigations indicated that the *DsbA-L* gene in local lung tissue plays a protective role against respiratory dysfunction through its antioxidant activity (Fig. 6). The results of this study suggest that the *DsbA-L* gene may play a role in protecting the respiratory function of the elderly, possibly through the increased systemic adiponectin functions secreted from adipocytes or systemic and/or local pulmonary antioxidant properties, although further studies are needed to verify the present findings.

## Methods

### Human studies

#### Subjects and study protocol

All subjects were Japanese participants in the elderly health screening program held by the Japanese Red Cross Kumamoto Health Care Center. A retrospective longitudinal analysis for 5.5 ± 1.1 years of follow-up was conducted among 318 subjects (age [range]: 67.5 ± 6.0 [56–84] years, BMI [range]: 22.7 ± 2.8 [15.8–35.2] kg/m^2^). Based on the subjects’ medical history, those with lung diseases other than airway obstruction, such as asthma, tuberculosis and lung cancer, were excluded from this study. The study complies with the Declaration of Helsinki and was approved by the ethics committees of the Faculty of Life Sciences at Kumamoto University and the Japanese Red Cross Kumamoto Health Care Center. All of the subjects provided their written informed consent prior to enrollment in the study. All analyses were performed in accordance with Ethical Guidelines for Epidemiological Research in Japan.

#### Measurements

The clinical information was recorded at each follow-up visit (i.e. at yearly intervals). The laboratory tests were performed using the standard methods of the Japan Society of Clinical Chemistry. The information regarding smoking habits and alcohol intake was obtained via face-to-face interviews with healthcare providers. Overweight and normal-weight status were defined as a BMI ≥ 23 kg/m^2^ and BMI < 23 kg/m^2^, respectively, based on the BMI cut-off point for identifying at-risk Asian Americans for type 2 diabetes screening^31^.

#### Genotyping

Genomic DNA was extracted from whole blood using a DNA purification kit (FlexiGene DNA kit; QIAGEN, Hilden, Germany). *DsbA-L* rs1917760 (-1308G > T) genotype was determined using a real-time TaqMan allelic discrimination assay (Applied Biosystems, Waltham, MA, USA) in accordance with the manufacturer’s protocol (assay no. C_11980950_10). To ensure the genotyping quality, we included DNA samples as internal controls, hidden samples of a known genotype, and negative controls (water).

#### Adiponectin measurements

To assess the associations of the *DsbA-L* genotype or respiratory function with APN multimerization, we measured the levels of total and HMW APN in serum at the endpoint of the observation period according to the methods that we reported previously^15^.

#### Measurement of oxidized HSA

To assess the association of the *DsbA-L* genotype and respiratory function with oxidative stress, we measured the redox state of HSA, which was reported to be a sensitive systemic oxidative stress marker^32,33^, in serum at the endpoint of the observation period according to our previously reported method^15^. The values of each of the albumin fractions (for human mercapto-albumin [HMA], human non-mercapto-albumin [HNA]1 and HNA2) were estimated by dividing the area of each fraction by the total area corresponding to HSA. A mixture of HNA1 and HNA2 was defined as oxidized HSA^15^.

### *In vitro* experiments

#### Reagents and antibodies

SP600125 was purchased from Enzo Life Sciences, Inc. (Farmingdale, NY, USA). Antibodies against phospho-ERK 1/2 (Thr-202/Tyr204), phospho-p38 MAPK (Thr-180/Tyr-182), phospho-JNK 1/2 (Thr183/Tyr185) and phospho-EGFR (Tyr1068) were purchased from Cell Signaling Technology, Inc. (Beverly, MA, USA). Monoclonal antibody against β-actin was purchased from Sigma-Aldrich Japan (Tokyo, Japan). Antibody against γ-tubulin was purchased from Santa Cruz Biotechnology, Inc. (Santa Cruz, CA, USA). MUC5AC antibody was purchased from Thermo Fisher Scientific Inc. (Waltham, MA, USA). Antibody against HO-1 was purchased from Abcam (UK).

#### Cell culture

16HBE14o-cells were generated as reported previously^34^ and grown in fibronectin/vitrogen/BSA-coated flask in MEM (Invitrogen, Inc., Carlsbad, CA, USA)^35^. These cells were maintained in MEM supplemented with 10% fetal bovine serum, 2% penicillin/streptomycin. Primary normal human bronchial epithelial (NHBE) cells were purchased from LONZA (Basel, Switzerland) and maintained according to the manufacturer’s instructions^35^. All cells were cultured in a humidified incubator at 37 °C and 5% CO_2_. ON-TARGETplus SMARTpool GSTK1 (DsbAL) siRNA (siDsbAL) (GE Healthcare Dharmacon, Inc., Lafayette, CO, USA) and control GL2 siRNA^36^ were transfected into 16HBE14o cells using Lipofectamine RNAiMAX (Thermo Fisher Scientific, Inc.). GSTK1 (DsbAL) plasmids in pcDNA3.1 + /C-(K)DYK (#1 and #2 correspond to NM_015917 and NM_001143679, respectively) were obtained from GenScript, Inc. (Piscataway, NJ, USA). These plasmids or pcDNA3.1 empty plasmid were transfected into NHBE cells using the Avalanche Transfection Reagent (EZ Biosystems, Maryland, USA), according to the manufacturer’s instructions.

#### A real-time quantitative reverse transcription (RT)-polymerase chain reaction (PCR) analysis

Quantitative RT-PCR was performed by protocols that were previously reported^37^. After reverse transcription, we performed real-time PCR with iQ5 (Bio-Rad) in a mix containing DNA polymerase and SYBR Green (PR820; Takara Bio, Inc., Shiga, Japan). The relative quantity of the target gene expression was normalized using human 18srRNA as the internal control and expressed as the relative quantity of target gene expression (fold induction). PCR amplification was performed in triplicate, and the reaction protocol included pre-incubation at 95 °C to activate Ex Taq HS for 30 s, amplification of 40 cycles set for 15 s at 95 °C, and annealing for 60 s at 60 °C. The sequences of primers used for real-time PCR are shown in Table 4.

**Table 4:** Primers used for quantitative RT-PCR.

Human mRNA	Forward primer	Reverse primer
18srRNA	5′- CGGCTACCACATCCAAGGAA-3′	5′- GCTGGAATTACCGCGGCT-3′
*MUC5AC*	5′-CAGCCACGTCCCCTTCAATA-3′	5′-ACCGCATTTGGGCATCC-3′
*DsbA-L*	5′-TCTGGAAAAGATCGCAACGC-3′	5′- GCCCAAAGGCTCCGTATCTG -3′
*HO-1*	5′-GGGAATTCTCTTGGCTGGCT -3′	5′- GCTGCCACATTAGGGTGTCT -3′

RT-PCR, reverse transcription-polymerase chain reaction; DsbA-L, disulfide bond-forming oxidoreductase A-Like protein; HO-1, heme oxygenase 1.

#### Slot and Western blotting analyses

Human bronchial epithelial cells were lysed in lysis buffer and subjected to SDS-PAGE and a Western blot analysis^38^. Blots were reacted for 2 h with monoclonal antibody diluted at 1:1000 and with the respective HRP-conjugated secondary antibodies diluted at 1:2000. After each antibody reaction, membranes were washed 3 times with 0.1% TBS-Tween, and blots were visualized with Super Signal West Pico chemiluminescence substrate (Thermo Fisher Scientific, Inc.). Images were captured by LAS-4000 (GE HealthCare Dharmacon, Inc.). The band intensity was quantified using the Multi Gauge software program (FUJIFILM, Inc., Tokyo, Japan). For the detection of MUC5AC protein secretion into the medium, slot blotting was performed as previously described^39^.

#### ROS detection

ROS production was detected using 2′,7′-dichlorodihydro-fluorescein diacetate acetyl ester (CM-H2DCFDA) (Invitrogen, Inc.) dye according to the manufacturer’s instructions. Pictures were taken using BioRevo BZ-9000 (Keyence, Inc., Tokyo, Japan). Exposure times were kept constant within each trial.

### Statistical analyses

Categorical variables were compared using Fisher’s exact test. Student’s *t*-test or a one-way analysis of variance were used to compare the differences in the continuous parametric valuables. Nonparametric data were analyzed using the Mann-Whitney U test or Kruskal-Wallis test. The associations of the *DsbA-L* genotype with the prevalence of FEV1/FVC < 70% and the longitudinal differences in the FEV1/FVC and %FEV1 were examined using logistic regression analyses with calculations of the odds ratios (ORs) and 95% confidence intervals (95% CIs) and using multiple linear regression analyses with calculations of the partial regression coefficients (Bs) and standard errors (SEs), based on the generalized estimating equations approach^40^. Multiple linear regression analyses were performed to compare the differences in the total, HMW APN, ratio of HMW/total APN and oxidized HSA among the *DsbA-L* genotypes, and the effects were adjusted for potentially confounding factors. Structural equation modeling was used to perform the pathway analysis assessing the effects of the *DsbA-L* genotype on the respiratory function in association with APN multimerization and oxidative stress. The goodness-of-fit on the structural equation modeling was evaluated based on the following criteria: GFI > 0.90, AGFI > 0.90, and RMSEA < 0.10. In order to examine the accuracy of the parameters of the structural equation model, bootstrap analysis was performed using 1000 replicated datasets generated by random sampling with replacement.

A value of *P* < 0.05 was considered to be statistically significant. Multiple comparisons were performed by Bonferroni’s correction, and P values < 0.05/number of comparisons made were considered to be statistically significant. The structural equation modeling process and the other statistical analyses were performed using the SPSS Amos software program (version 23.0; IBM Japan, Inc., Tokyo, Japan) and the SPSS software package (version 23.0; IBM Japan, Inc.), respectively.

## Supplementary information

Supplementary Information.

## Data Availability

The datasets generated and/or analyzed during the current study are not publicly available due to individual privacy but are available from the corresponding authors on reasonable request.
