# Supplementary material for: The DsbA-L gene is associated with respiratory function of the elderly via its adiponectin multimeric or antioxidant properties
Source: Sci Rep. 2020 Apr 6;10:5973. doi: 10.1038/s41598-020-62872-5 (PMC7136289; doi:10.1038/s41598-020-62872-5)

Supplementary Information

The *DsbA-L* gene is associated with respiratory function of the elderly via its adiponectin multimeric or antioxidant properties

Kentaro Oniki, Hirofumi Nohara, Ryunosuke Nakashima, Yui Obata, Narumi Muto, Yuki Sakamoto, Keiko Ueno-Shuto, Tadashi Imafuku, Yu Ishima, Hiroshi Watanabe, Toru Maruyama, Koji Otake, Yasuhiro Ogata, Mary Ann Suico, Hirofumi Kai, Tsuyoshi Shuto, Junji Saruwatari

Supplementary Table S1. Longitudinal associations of the combination of *DsbA-L* genotype and weight status with the values of FEV1/FVC and %FEV1 and the prevalence of FEV1/FVC <70% in the multivariable regression models based on the generalized estimating equations approach

Weight status	*DsbA-L* genotype	FEV1/FVC (%)			%FEV1 (%)			FEV1/FVC <70%		
		B*	SE	*P*	B*	SE	*P*	OR*	95% CI	*P*
Normal-weight	G/G	0			0			1		
	G/T	-2.805	0.804	<0.001†	-3.073	2.143	0.152	3.227	1.266 - 8.230	0.014
	T/T	0.343	1.743	0.844	-2.258	5.299	0.670	0.390	0.049 - 3.086	0.372
Overweight	G/G	0			0			1		
	G/T	-0.798	0.928	0.390	-0.690	2.426	0.776	1.609	0.595 - 4.351	0.348
	T/T	-4.638	1.968	0.018	-12.550	4.339	0.004†	5.702	1.335 - 24.357	0.019

* Adjusted by age, gender, BMI, smoking status. † Statistical significance remained following the use of Bonferroni's correction. DsbA-L, disulfide bond-forming oxidoreductase A-like protein; BMI, body mass index; %FEV1, percent predicted forced expiratory volume in 1 second; OR, odds ratio; CI, confidence interval; SE, standard error.

Supplementary Table S2. The bootstrap analysis of the structural equation model

			Estimates		Bootstrap analysis			
			β	P	Mean	SE	95% CI	P
Oxidized HSA	←	Overweight	0.135	0.015	0.134	0.055	0.022, 0.241	0.020
HMW/total APN	←	Overweight	-0.142	0.009	-0.145	0.056	-0.257, -0.037	0.010
Oxidized HSA	←	*DsbA-L* T allele	0.144	0.009	0.144	0.054	0.041, 0.250	0.012
HMW/total APN	←	*DsbA-L* T/T genotype	-0.115	0.039	-0.114	0.050	-0.211, -0.020	0.015
FEV1/FVC <70%	←	*DsbA-L* T allele	0.119	0.025	0.120	0.054	0.018, 0.235	0.019
FEV1/FVC	←	*DsbA-L* T allele	-0.125	0.016	-0.126	0.052	-0.228, -0.026	0.014
%FEV1	←	*DsbA-L* T/T genotype	-0.104	0.041	-0.102	0.047	-0.192, -0.008	0.032
FEV1/FVC <70%	←	Ever-smoking status	0.106	0.015	0.107	0.046	0.020, 0.197	0.010
%FEV1	←	Ever-smoking status	-0.175	<0.001	-0.175	0.050	-0.269, -0.077	0.003

β, partial regression coefficient; SE, standard error; HSA, human serum albumin; HMW, high molecular weight; APN, adiponectin; FEV1: percent predicted forced expiratory volume in 1 second; FVC, percent predicted forced vital capacity; DsbA-L, disulfide bond-forming oxidoreductase A-like protein.

Supplementary Figure S1. Full unedited gel for Figure 4-C

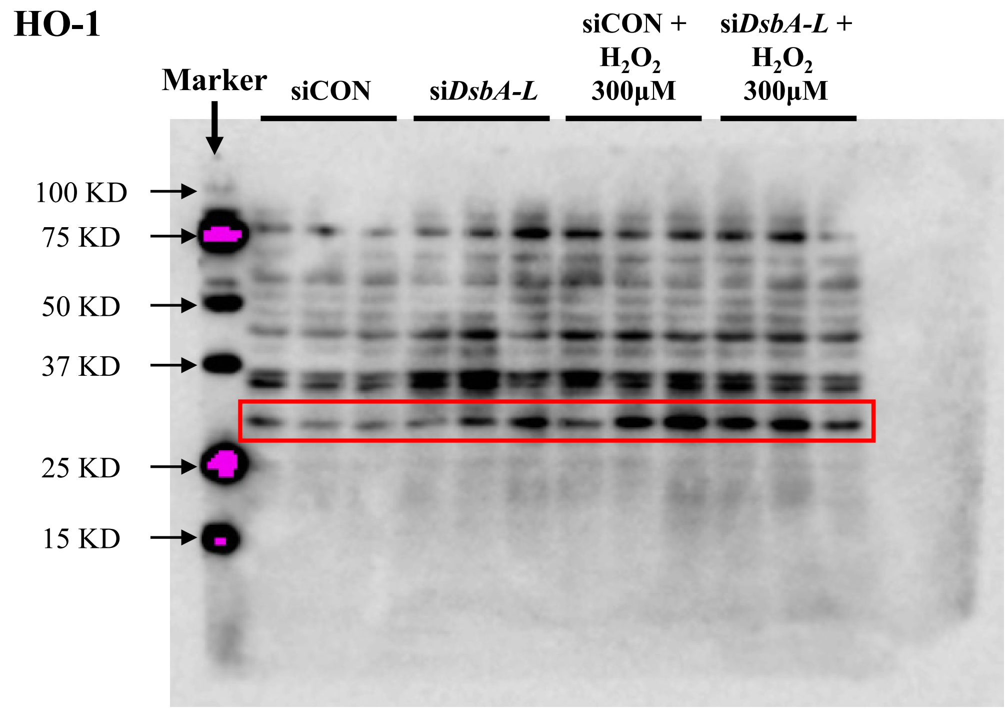

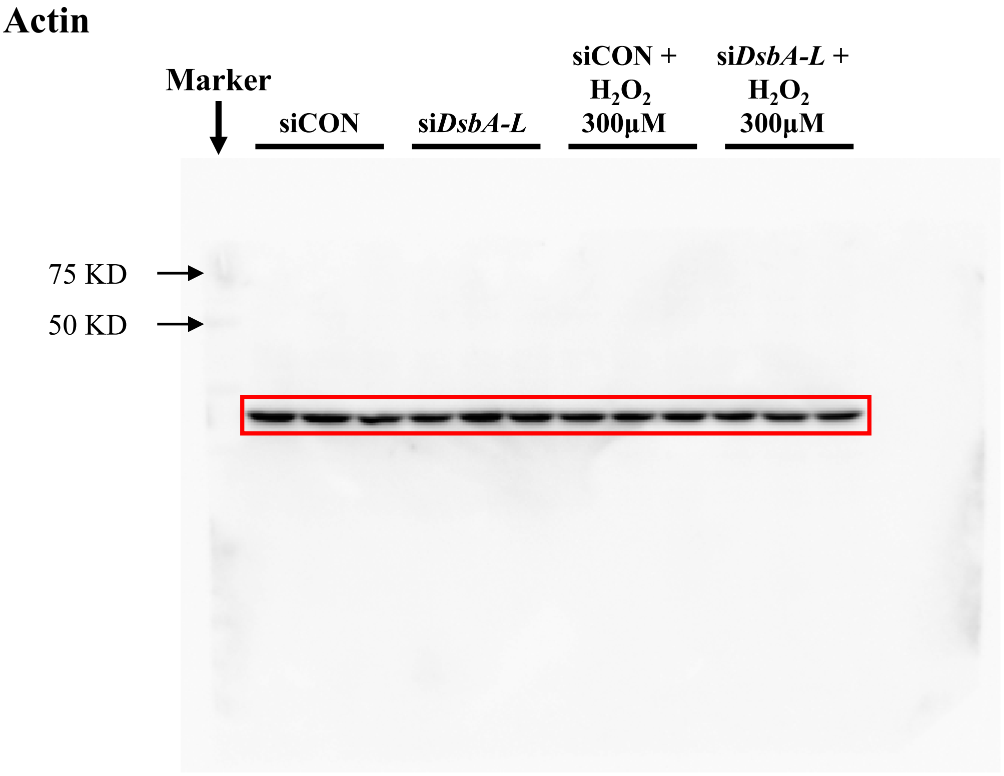

Supplementary Figure S2. Full unedited gel for Figure 4-E

DsbA-L

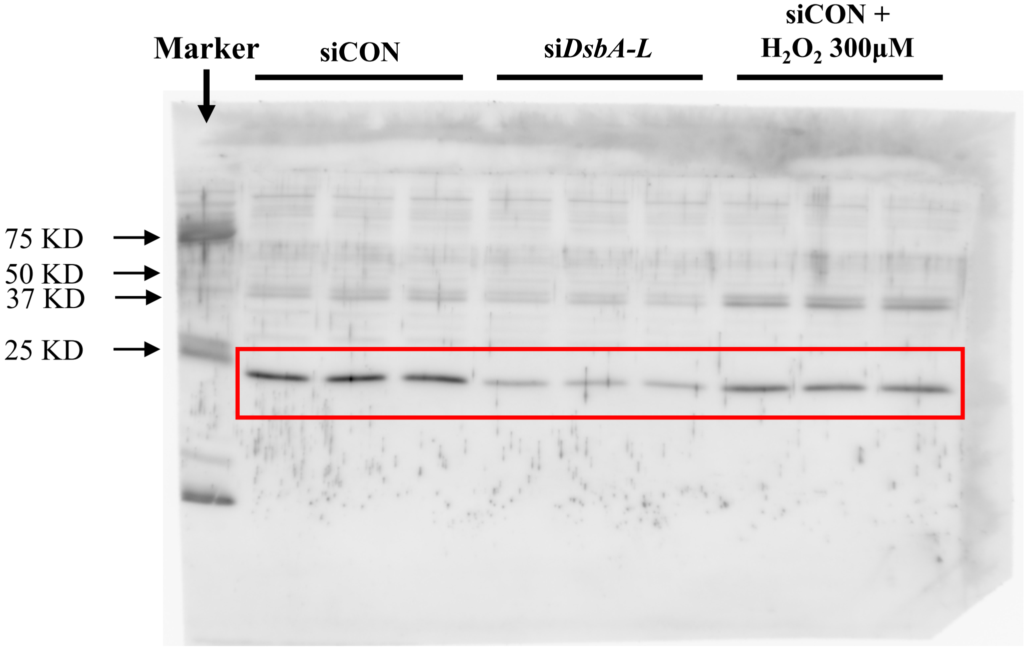

γ-Tubulin

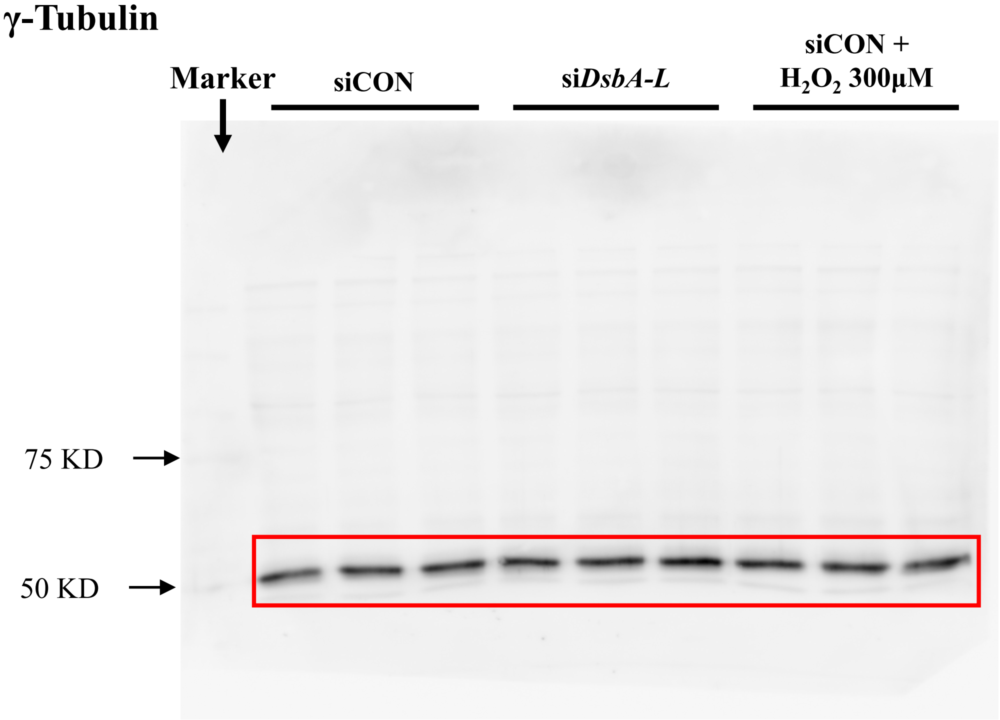

Supplementary Figure S3. Full unedited gel for Figure 4-F

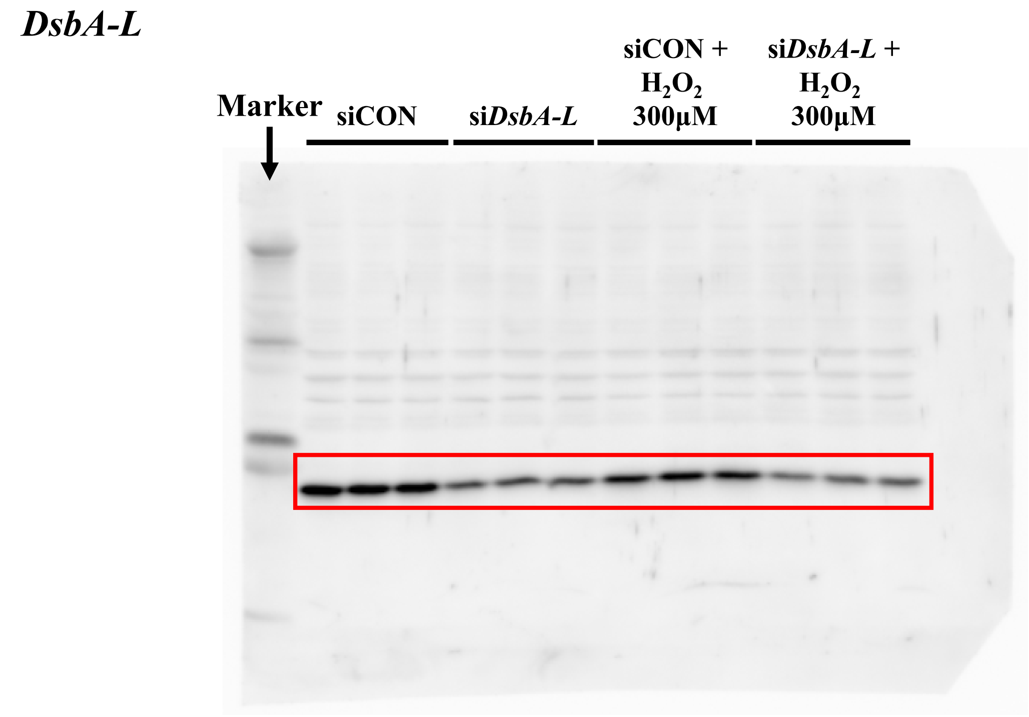

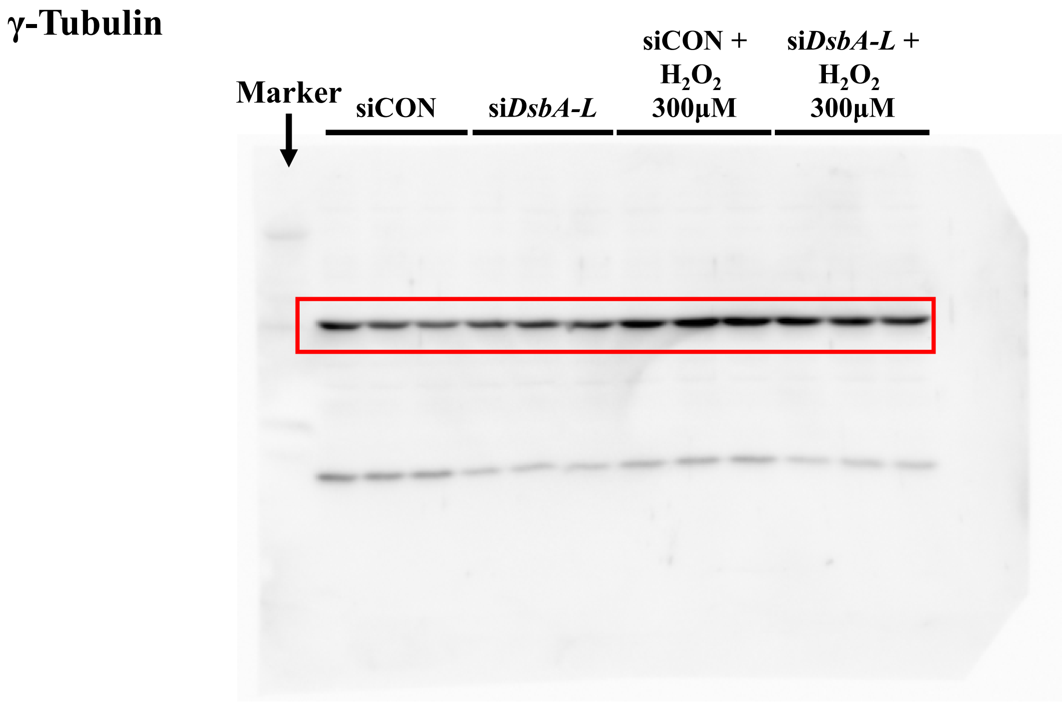

Supplementary Figure S4. Full unedited gel for Figure 5-B

MUC5AC protein

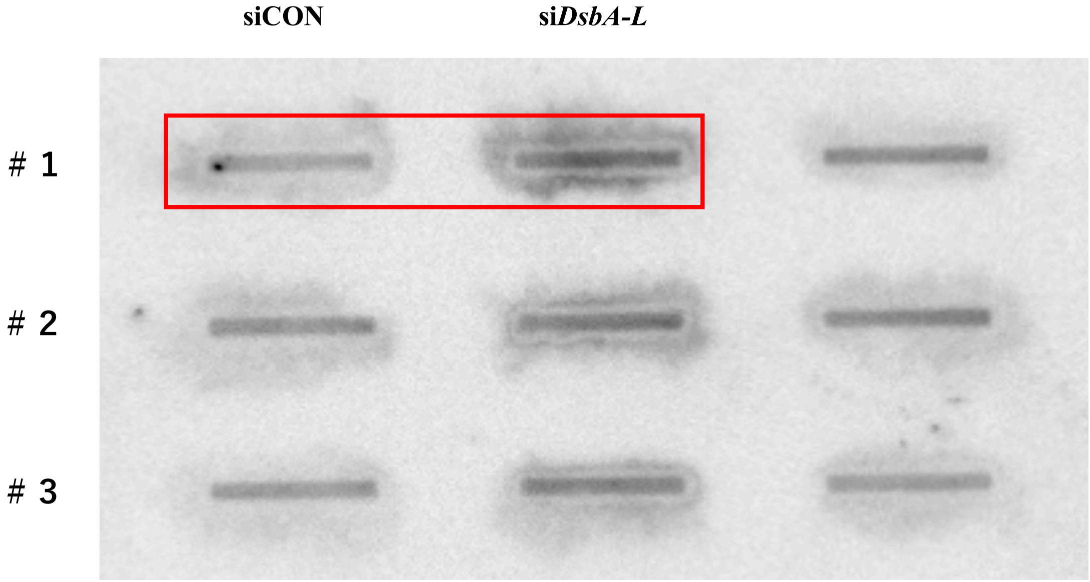

Supplementary Figure S5. Full unedited gel for Figure 5-C

p-JNK

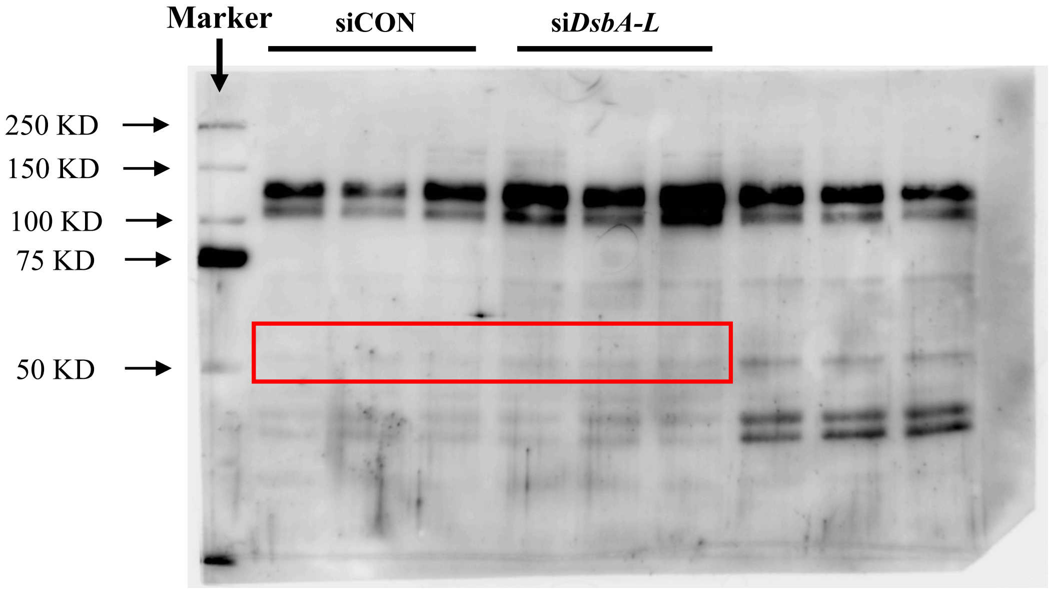

p-p38

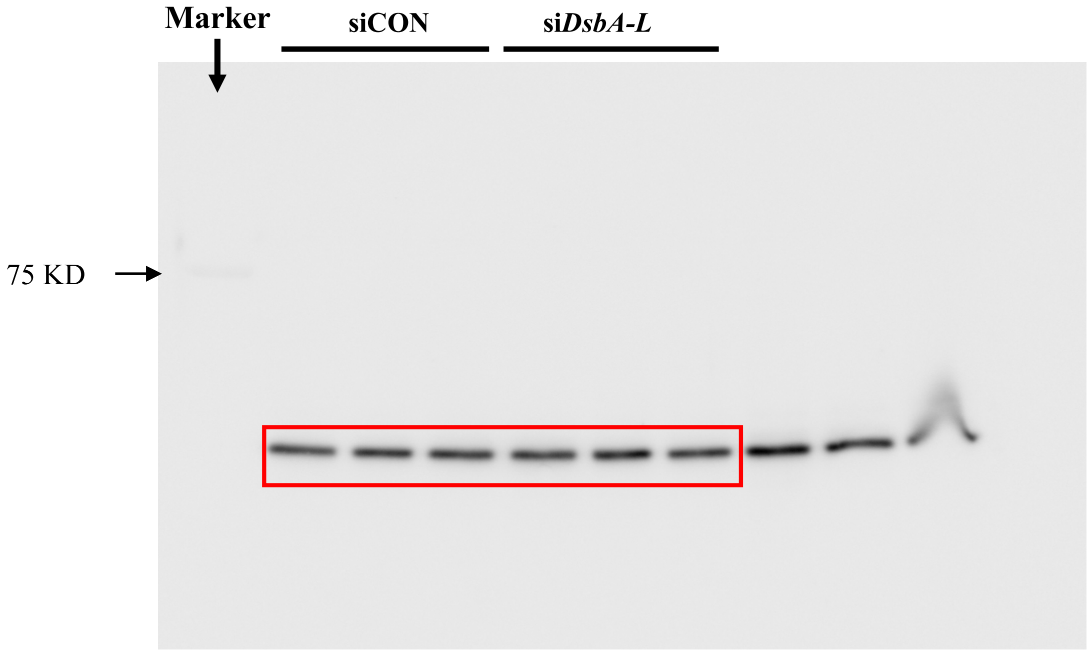

Supplementary Figure S6. Full unedited gel for Figure 5-C

p-ERK

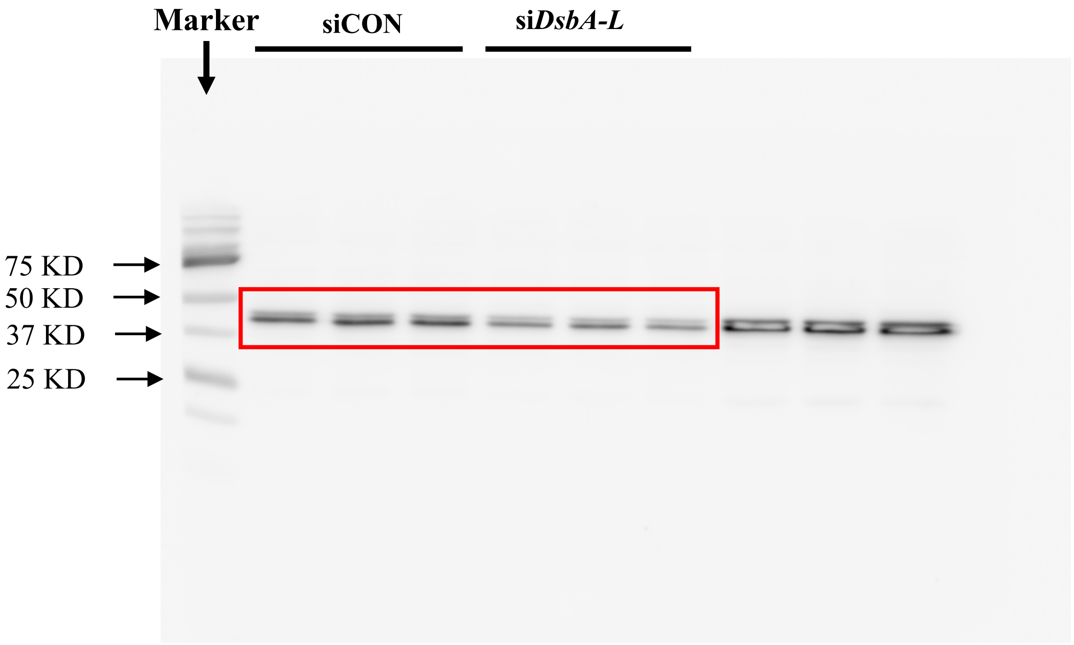

p-EGFR

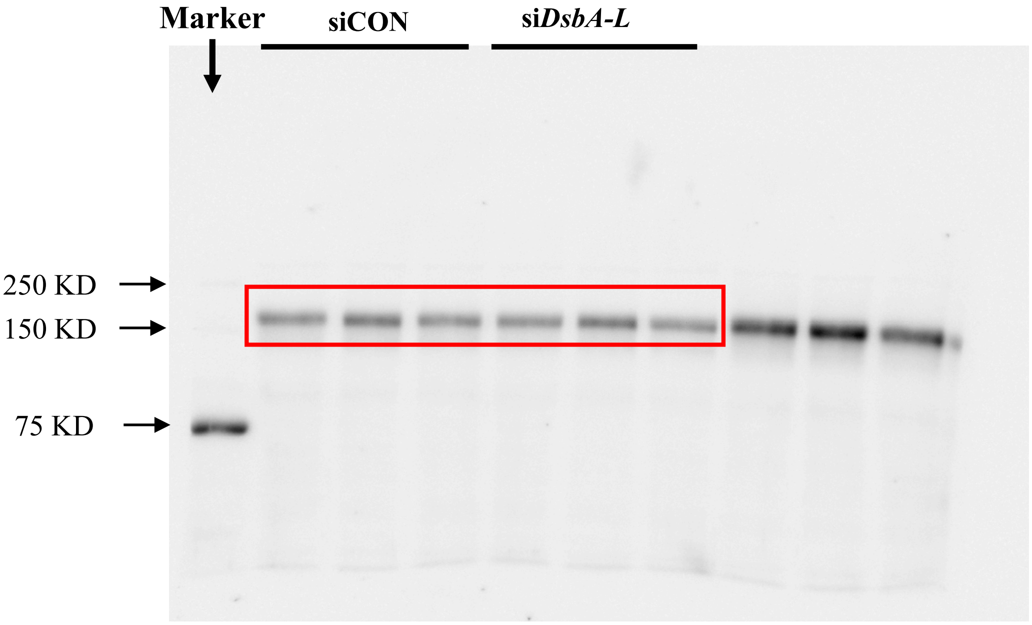

Supplementary Figure S7. Full unedited gel for Figure 5-C

Actin

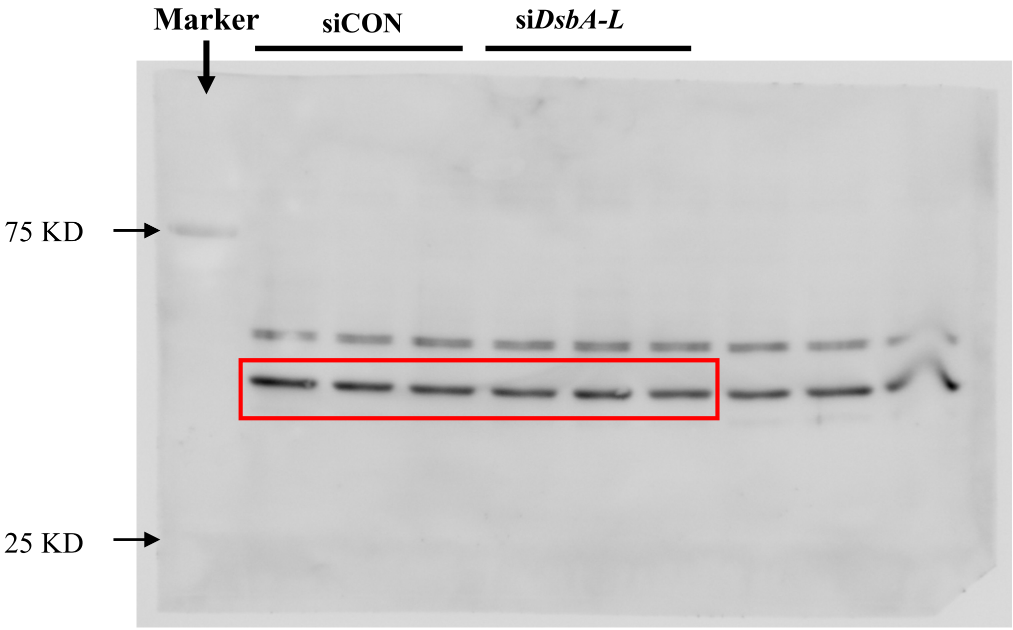

Supplementary Figure S8. Full unedited gel for Figure 5-D

p-JNK

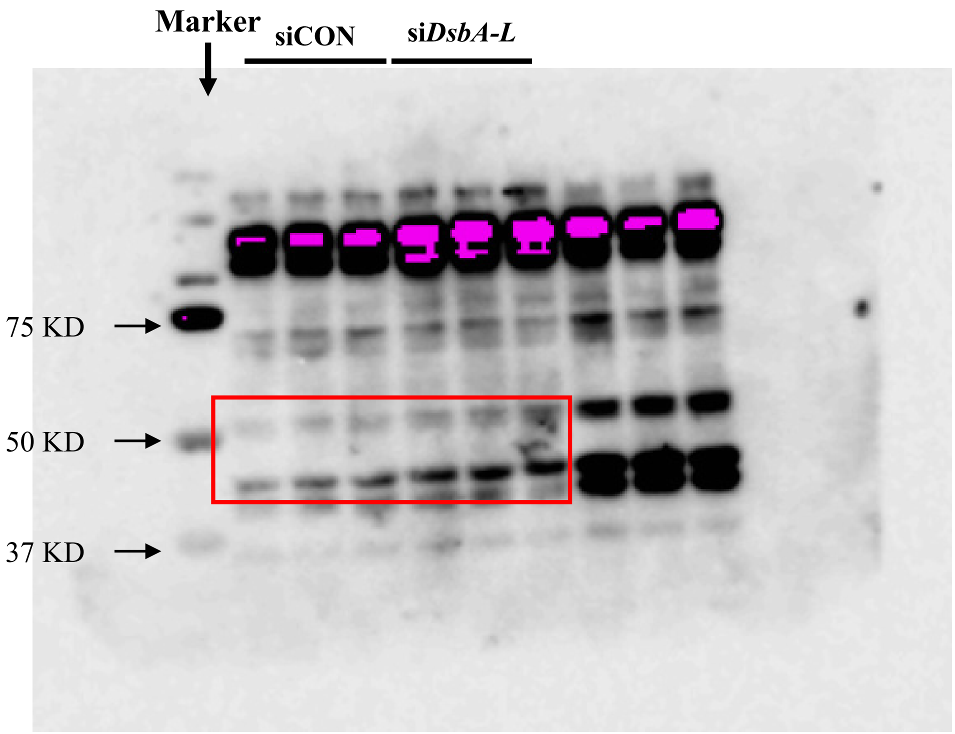

Actin

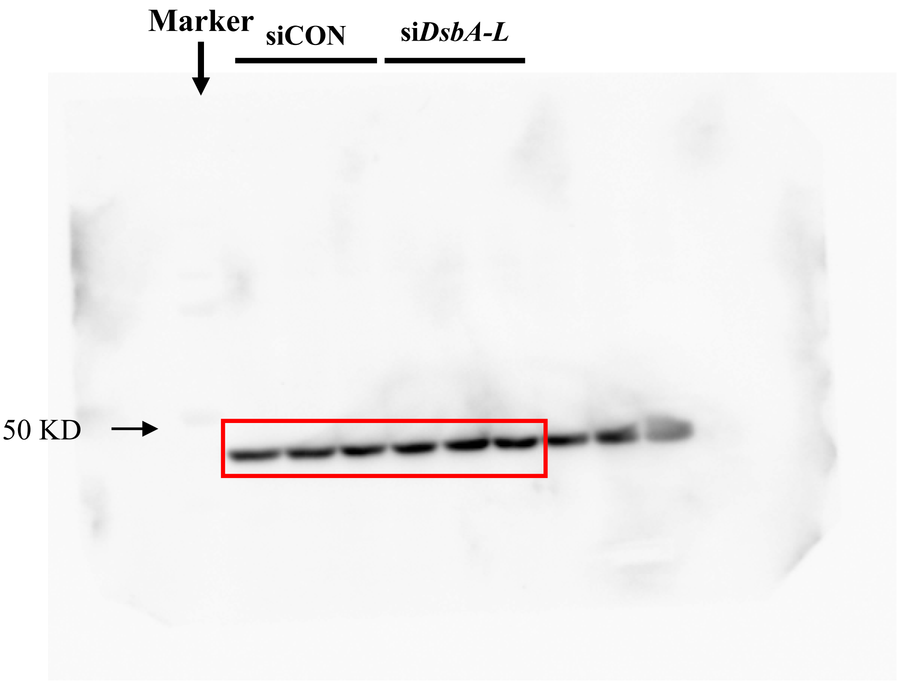

Supplement: Supplementary file 1 — Supplementary Information. [file 41598_2020_62872_MOESM1_ESM.pdf]
